# An Improved Impact Source Locating System Using FBG Rosette Array †

**DOI:** 10.3390/s19163453

**Published:** 2019-08-07

**Authors:** Bo-Lian Chen, Chow-Shing Shin

**Affiliations:** Department of Mechanical Engineering, National Taiwan University, No.1, Sec. 4, Roosevelt Rd, Taipei 10617, Taiwan

**Keywords:** Fiber Bragg grating, FBG array, impact source localization, directional sensitivity, FBG rosette, impact monitoring, structural health monitoring

## Abstract

For structures vulnerable to foreign object impact damages, it would be desirable to detect and locate any occurrence of such impacts. This can be achieved by monitoring the stress waves generated by an impact together with certain source localization algorithms. Being small, electromagnetic influence immune and durable, Fiber Bragg grating (FBG) sensors are advantageous for this task. One drawback of FBGs for this purpose is their uneven directional sensitivity, which limits its localization ability to within 50° on either side of the fiber axis. Beyond this range, the signal is too weak and masked by noises and the location errors increase abruptly. Two approaches have been tested on a 0.8 m × 0.8 m × 6 mm plate for possible improvement on the system accuracy: firstly, an interrogation scheme with stronger light source intensity and steeper edge filter is employed to enhance the signal-to-noise ratio and system sensitivity; secondly, rosettes with two orthogonal FBGs are cascaded together to replace single FBGs to alleviate the directional sensitivity problem. It was found that a four-fold increase in signal to noise ratio contributed by stronger light source does improve the location accuracy, but only marginally. For the rosette approach, the relative positions of the Bragg wavelength of the FBGs and the light source spectrum are crucial to accuracy. Three different wavelength configurations have been tested and the reasons for their success or failure are discussed. It was shown that with an optimal wavelength configuration, the rosette array can virtually extend the good location accuracy to all over the plate.

## 1. Introduction

Some engineering structures are vulnerable to low velocity impact damages. One example is underground pipelines, which may occasionally be dented or gouged by the impact of blunt excavation tools [1,2]. Another example is polymeric composite structures such as aircraft fuselage and wind turbine blades, which may suffer impact of foreign objects like bird strikes, hailstorms, or dropped equipment [3]. Such impact may induce internal damages that may continue to develop under fluctuating service loading and adverse environment and can lead to catastrophic failures eventually.

Although non-destructive examination techniques for the detection of the above internal damages are available, carrying out an examination over the entire structure to look for the occurrence of such defects is highly time consuming and uneconomical. If the occurrence and the location of an impact event are known, a detailed examination effort can be concentrated on the affected areas. Impact events on plate-like structures generate Lamb waves [4], which can propagate over long distances [5]. By detecting and localizing the origin of the Lamb waves, the occurrence and location of an impact can be identified. Conventionally, piezoelectric sensors have been used for this purpose [6,7,8,9,10,11,12,13]. Owing to their small size and weight, immunity to electromagnetic noises, good corrosion, fatigue damage resistances, and multiplexing capabilities, Fiber Bragg gratings (FBGs) have become popular sensing elements for structural health monitoring (SHM). Several pilot researches demonstrated FBG’s capability to detect stress waves [14,15] and a number of ways to localize impact events by FBG sensors have been proposed [16,17,18,19,20,21,22]. A number of techniques, which have been employed as the foundations of source location algorithms, are briefly reviewed below.

Time difference of arrival (TDOA) methods are based on measuring the differences of arrival times from the source to multiple sensors. A difference in signal arrival time between any two sensors in combination with their coordinates and wave speed can define a hyperbola. The intersection of multiple hyperbolas gives an estimate of the source location [23]. Triangulation is the most representative algorithm of TDOA methods and is applicable to locate impacts on isotropic materials with known and unknown wave speed [5,6]. With an array consisting of four FBG sensors, good impact localization accuracy was obtained when the impact position was within the envelopment of certain angular boundaries around the sensors due to FBG’s insensitivity at certain directions [16]. The method can be generalized to anisotropic materials by knowing the wave velocity profile, which is the function of wave speed in terms of wave propagating direction, and has been demonstrated in carbon fiber reinforced composite [8]. It was found that FBG sensors led to larger errors of impact localization compared with PZT sensors, owing to FBG’s uneven directional sensitivity [17].

Transient excitation, like acoustic waves, can have more sparse time-frequency representations by wavelet transform (WT). Through WT analysis, it is possible to distinguish the arrival times of different kinds and different modes of stress waves [9]. Impact localization could be achieved by leveraging energy-rich waves [12] to make TDOA methods more robust to noises. Also, impact sources could be localized with fewer sensors by using the arrival times of different modes of stress waves [9,10]. It was found that the wavelet transform of a FBG response had detectable energy up to 180kHz when the direction of incidence wave was along FBG’s longitudinal axis, while it dropped to 130kHz when the angle of incidencewas60° with the FBG axis [18].

Beamforming is a signal processing technique in combination with microphone array measurements for sound source localization [11] and was recently applied for impact source localization [12,13]. Based on specific configurations of sensor array and weighting functions, the superposition of all the sensor waveforms was used to estimate the possible impact locations. Direct measurement of signal arrival times, as required by TDOA methods, was not needed. The delay-and-sum (DAS) [19] and adaptive minimum variance (MV) beamforming [19,20] algorithms were used to locate acoustic sources by FBG sensors. It was found that FBG’s directional sensitivity undermined the performance of both beamforming methods, with the accuracy of the DAS algorithm affected more than the MV algorithm [20].

One localization method not influenced by FBG’s directionality imitates strain gauge rosettes. The FBG rosette, consisting of three individual FBG sensors with different orientations at a position, was first proposed for evaluating the principal stresses and their directions in a static stress field [24]. Assuming the propagating directions of the stress wave and its induced principal stress are the same, the intersection of two principal stress directions estimated by two FBG rosettes could approximate the impact location [21,22]. The drawbacks of this method are that each FBG in a rosette has to be interrogated and calibrated for strain sensitivity individually. Moreover, this scheme is not applicable to anisotropic plates, as the above-mentioned assumption does not hold [25].

In summary, the directional sensitivity of FBGs not only degrades the accuracy of the signal arrival time detection but also causes permanent loss of contents in the time and frequency domains. FBG’s directionality could be improved physically by adding transducer-like mechanisms [26,27] or using two independent FBGs at each sensor position, with each FBG interrogated individually [17,27]. However, special geometric requirements on thickness to accommodate the mechanism would limit the former’s applications, and the latter method would halve the light intensity, leading to degraded resolution, and double the needed data acquisition channels.

**Figure 1:**
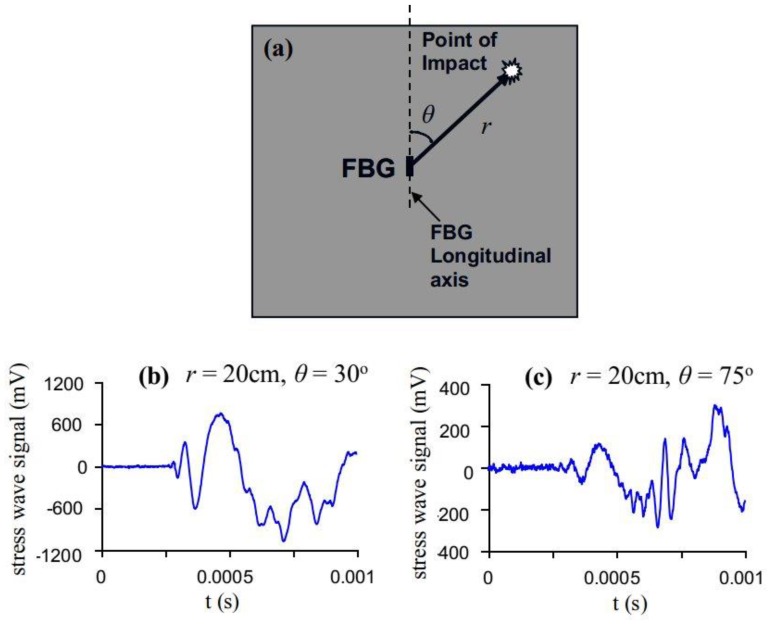
(**a**) Experimental setup for evaluating sensitivity under different (*r*, *θ*)’s; (**b**) received waveform for impact at *r* = 20cm, *θ* = 30°; (**c**) received waveform for impact at *r* = 20cm, *θ* = 75°.

In a preliminary work using an energy modulating interrogation scheme [16], it was found that the localization ability of a 4-FBG array is limited by (i) signal noise and (ii) the directional sensitivity of the sensor. The latter phenomenon is exemplified in Figure 1, where a fixed energy impact was conducted at a distance of *r* = 20 cm from the FBG while the angle *θ* varied. Signal waveforms received from various *θ* were recorded. Impacts that lie close to the FBG axis give large amplitude signals with well-defined initial peaks and valleys (Figure 1b). As the off-axis angle *θ* increases, signal amplitude decreases to such an extent that the initial peaks and valleys are indistinguishable from the background noise (Figure 1c). In the current work, we look at the possibility of compensating this loss in sensitivity by either significantly increasing the intensity of the light source and enhancing the sensitivity of the modulating scheme or employing a cascaded pair of orthogonal FBGs to do away with FBG’s insensitivity at certain directions.

## 2. Experimental and Analysis Procedures

### 2.1. Impact Testing and Sensor Layout

**Figure 2:**
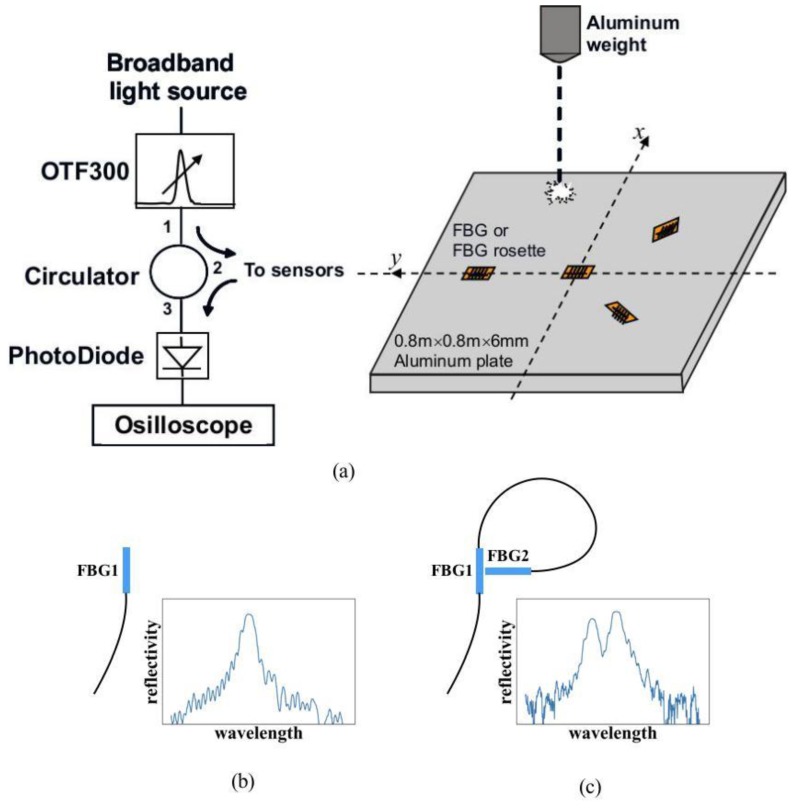
(**a**) Sensor layout and the Amplified Spontaneous Emission (ASE) light source scheme; (**b**) single Fiber Bragg grating (FBG) and its spectrum; (**c**) rosette configuration and typical spectrum.

**Figure 3:**
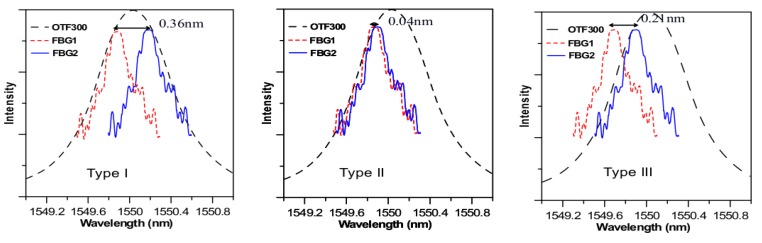
Layout of the spectra of the two Fiber Bragg grating (FBGs) in respect to the edge filter spectrum of OTF300: (**a**) Type I Rosette; (**b**) Type II Rosette; (**c**) Type III Rosette.

Impact localization testing was carried out on a 0.8 m × 0.8 m ×6 mm uniform aluminum plate. An array of four sensors was glued on the plate at (0,0.225), (0,0), (−0.159, −0.159), and (0.159, −0.159), where (0,0) is the plate center (Figure 2a). The sensors may be single Fiber Bragg grating (FBG) or two orthogonal FBGs cascaded together to form a rosette. In the single FBG case, the FBGs at (0,0.225) and (0,0) were aligned with the y axis, while those at(−0.159,−0.159) and (0.159,−0.159) were aligned with the ± 45° diagonal directions of the plate. In the rosette case, one of the two FBGs has the same orientation as that in the single FBG configuration. A second FBG is attached orthogonally and with its longitudinal axis crossing the first FBG’s center point. The physical layouts of a single FBG and a Rosette, together with their typical reflective spectra, are illustrated schematically in Figure 2b,c). Three different rosette configurations (designated Type I, II, and III Rosettes respectively), each with the same physical layout as in Figure 2c, but a different relative Bragg wavelength arrangement, as shown in Figure 3, have been tested.

A grid with 5 cm spacing was marked on the plate. Impact events were made by dropping a blunt-headed 70 g aluminum cylinder from a fixed height of 30 cm onto each grid point. To make use of symmetry, impact was made to half of the plate.

All FBGs used have peak wavelengths between 1551 and 1552 nm, a reflectivity of about 99%, and a 3 dB bandwidth of about 0.14 nm. The FBGs were fabricated by side writing on Ge-B co-doped single mode photosensitive fibers. When the FBG receives a stress wave signal, its characteristic Bragg wavelength will shift and the amount of shift was interrogated, with two different intensity demodulation schemes detailed below.

### 2.2. Fiber Bragg grating (FBG) Interrogating Schemes

#### 2.2.1. Amplified spontaneous emission Light Source with Tunable Edge Filter

One interrogating scheme employed an amplified spontaneous emission (ASE) light source (Figure 2a). The broadband ASE light was modified with a tunable edge filter (Santec OTF300-003-S3) with ~20 dbm monotonic intensity variation over a 1.2 nm. The edge of the filter was tuned to fall on the Bragg wavelength (FBG1 spectrum in Figure 3c). The overlapping part of the filter and Fiber Bragg grating (FBG) spectra represents the light reflected from the FBG. Any shift in the Bragg wavelength alters the amount of this overlap, thus changing the reflected light intensity from the FBG. The light source was split to supply four sensors. The modulated light outputs were converted to voltages through photodiodes and recorded with a 4-channel digital storage oscilloscope. Both the single FBG sensors and the FBG rosettes are interrogated with this scheme.

#### 2.2.2. Tunable Fiber Ring Laser

**Figure 4:**
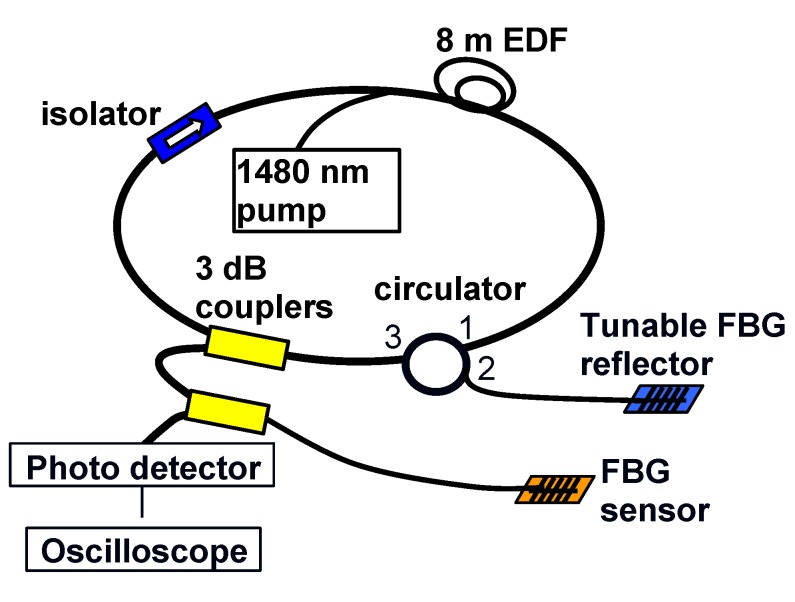
Interrogation scheme based on a ring laser.

Figure 4 shows another interrogation scheme using a fiber ring laser. Pumped with 1480 nm light, the light in the 1550 nm wavelength region, which is reflected by a Fiber Bragg grating (FBG) reflector mounted on a mechanical stretching stage, is amplified by a section of 8m erbium-doped fiber (EDF) and circulates through the ring cavity. The lasing wavelength was tuned to fall on the edge of the FBG sensor. This results in a more intense light source that will boost the signal intensity. Moreover, because the edge slope of the FBG reflector is steeper than the tunable edge filter in the amplified spontaneous emission (ASE) scheme, the system will have better sensitivity to any shift in the Bragg wavelength of the sensing FBGs.

#### 2.2.3. Impact Source Locating Algorithm

**Figure 5:**
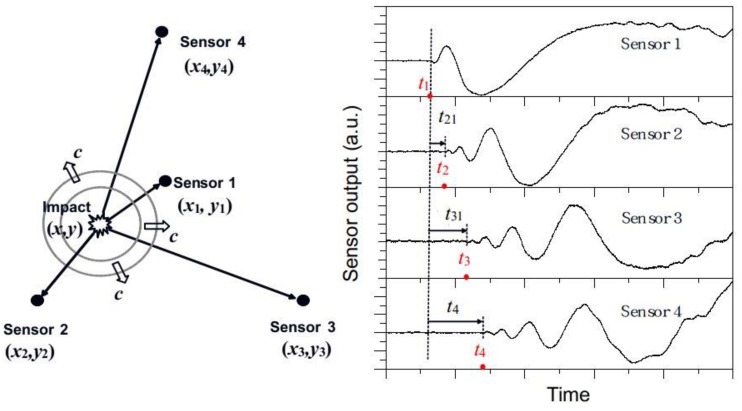
Nomenclature of the four-sensor algorithm to evaluate the impact location.

Figure 5 is a schematic explanation of the time difference of arrival method employed in this work using four sensors. The coordinates of an impact (*x*,*y*) and the wave speed *c* can be estimated by solving the following non-linear equations based on the triangulation scheme:

(1) $$\begin{array}{c} \sqrt{{\left(x-x_{2}\right)}^{2}+{\left(y-y_{2}\right)}^{2}}=\sqrt{{\left(x-x_{1}\right)}^{2}+{\left(y-y_{1}\right)}^{2}}+ct_{21}\\ \sqrt{{\left(x-x_{3}\right)}^{2}+{\left(y-y_{3}\right)}^{2}}=\sqrt{{\left(x-x_{1}\right)}^{2}+{\left(y-y_{1}\right)}^{2}}+ct_{31}\\ \sqrt{{\left(x-x_{4}\right)}^{2}+{\left(y-y_{4}\right)}^{2}}=\sqrt{{\left(x-x_{1}\right)}^{2}+{\left(y-y_{1}\right)}^{2}}+ct_{41} \end{array}$$

where *t_i_*_1_ is the time difference between the first arrival points of stress waves to the sensor *i* and 1.*t_i_*_1_’s can be derived from the oscilloscope recorded signals. Each arrival time was obtained by first filtering the waveform with a digital band pass filter with cutoff frequencies of 1 kHz and 100 kHz. A proper length of the leading part of the filtered waveform excluding the impact signal was then chosen to compute the average noise level. The first point where the waveform deviates 50% beyond this average noise level is taken as the first arrival point.

## 3. Results and Discussion

### 3.1. Signal Strength of the Two Interrogation Schemes

**Figure 6:**
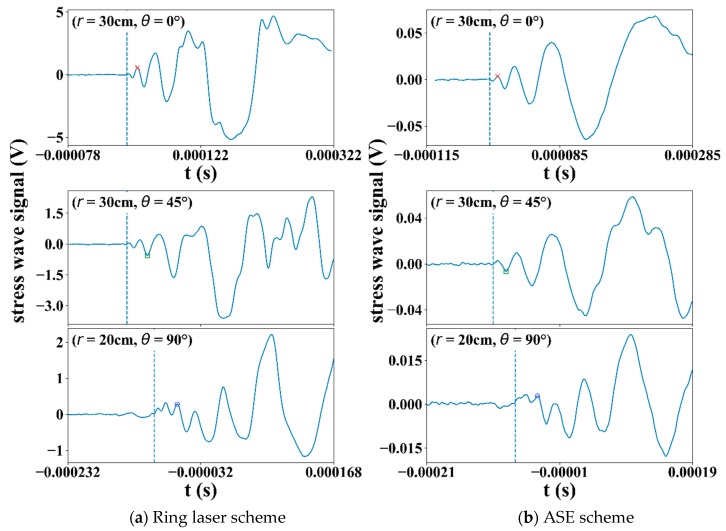
Stress wave received using: (**a**) the ring laser scheme; (**b**) the amplified spontaneous emission scheme when impacts were made at different (*r*, *θ*)’s. Note that for each scheme, signals for *θ* = 45° and 90° were from the same impact event.

The plate with FBG array in Figure 2 was hooked up with the Amplified Spontaneous Emission (ASE) light source scheme and the ring laser scheme in turn. The aluminum weight was dropped onto chosen positions to produce impact events at different (*r, θ*)’s, using the nomenclatures of Figure 1a. Figure 6 compares typical signals received for *θ* equal to 0°, 45°, and 90°. The impact signals for *θ* = 45°and *θ* = 90°were logged from two different FBGs with the same impact events and so have= different *r*’s but a common time axis. From the scale on the *y*-axis, it is clear that the signal from the ring laser scheme is about ten times stronger than that from the ASE scheme because of the former’s significantly higher light source intensity. For each *θ*, the first significant peaks in the signals from both schemes were marked by an arrow pointing to the same symbol on the waveforms. The amplitude of these peaks divided by the average amplitude of a proper length of the leading part of the corresponding waveforms gave signal-to-noise ratios (SNR). The SNRs of the ring laser scheme were 3.28 times, 2.95 times, and 4.33 times larger than that of the ASE light source scheme for *θ* equal to 0°, 45°, and 90°, respectively. Dotted lines in the figure indicate the first arrivals of the impact signals, deduced using the method stated above. For *θ* = 90°, finer details that immediately follow the first arrival is not as well-defined as that in 0° and 45°.Note that the distance between the impact point and the FBG was 20 cm for the *θ* = 90°case, yet it was 30 cm for the *θ* = 45°case. The loss of the initial fine details masks the definition of the first arrival time, and this explains why the signals for *θ* = 90°apparently took longer to arrive, although the impacts were closer to the FBG. The above phenomena exist in both schemes and the significantly raised source intensity and SNR of the ring laser scheme do not seem to offer marked improvement.

### 3.2. Angular Sensitivities for Different Fiber Bragg Grating Rosette Configurations

The rosettes were introduced with the hope that the synergic effect of two orthogonal Fiber Bragg grating Gratings (FBGs) may alleviate the angular dependency of the sensitivity. The modulated energy output in any instance from each FBG will depend on the relative position of its Bragg wavelength to the filter spectrum, the polarity, and the amplitude of the arriving stress waves. As the FBGs were connected in series, their respective modulated energy output may strengthen or cancel each other. Since the above factors interact in a complex manner, some preliminary screening to determine the optimum configuration in terms of the combination of Bragg wavelengths of the two FBGs and their relative positions with the filter spectrum is needed. Three configurations (designated Type I, II, and III Rosettes, respectively, with a relative Bragg wavelength arrangement shown in Figure 3), were tested for directional sensitivities using an arrangement illustrated above in Figure 1. In Type I Rosette, the peaks of the two FBG spectra were separated from each other by ~0.36 nm so that there was one FBG spectrum close to each edge of the filter spectrum (Figure 3a). Type II Rosette had the two FBG spectra nearly overlapped; both are close to one of the filter spectrum edges (Figure 3b). Type III Rosette has the two FBG spectra separate from each other by ~0.21 nm lying on either side of the same filter spectrum edge (Figure 3c). The FBG1 referred to in Figure 3 in each rosette configuration is along the *θ* = 0° direction. At each angle *θ* tested, the impact signal was first received by using FBG1 alone and then with the respective rosettes.

**Figure 7:**
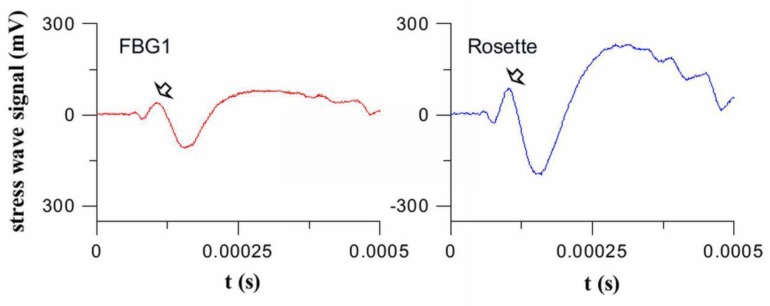
Signal waveforms received by Fiber Bragg grating (FBG 1) and the Type III Rosette when impact was made at *r* = 20 cm and *θ* = 45°. The peaks indicated by an arrow are used for signal strength comparison.

**Figure 8:**
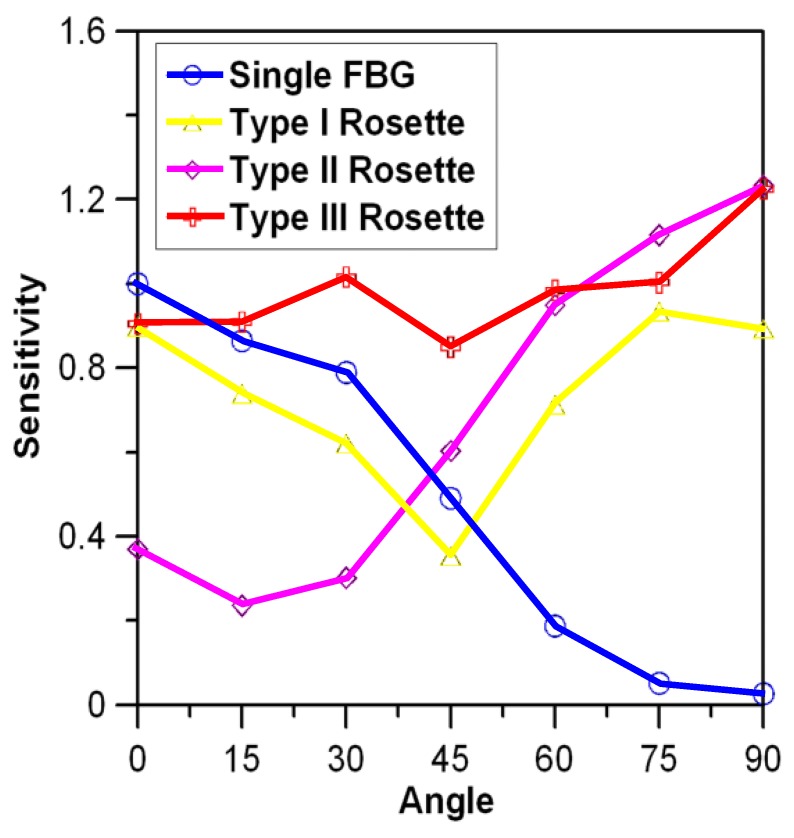
Sensitivity comparison between a single Fiber Bragg grating (FBG1) and different FBG rosette configurations.

Figure 7 shows a typical output from FBG1 and the Type III Rosette when *θ* = 45°. The amplitude of the first significant peak (pointed with an arrow in Figure 7) from each received waveform was used for rosette angular sensitivity comparison. These peak sensitivities were divided by the peak sensitivity of FBG1 when impact was made at *θ* = 0° for normalization. The normalized signal strengths from each rosette configuration were compared against those from the single FBG1 for various *θ*’s in Figure 8.

The directional sensitivity of a single FBG decreases as *θ* increases from 0° to 90°. In the rosettes, at *θ* = 45°, the signals received by both FBGs are basically of the same intensity and polarity with each other. That means the two FBG spectrum will move together as a whole by the same amount in the same direction. Referring to Figure 3a, for Rosette I, this phenomenon will lead to an increase in the output energy from one FBG while leading to a decrease from the other. Thus, the two have a tendency to cancel out and give a minimum output here. As *θ* deviates from 45°, the movements of the two spectra are still in phase, but that from the FBG with the smaller off-axis angle is getting increasingly larger and dominates the modulated output over the other. The above canceling effect is still in place but will decrease. This explains why the angular sensitivity of Type I Rosette is similar to, but slightly lower than, that of the single FBG1 for *θ* ≤ 45°.It has a minimum at *θ* = 45° where the canceling effect is most significant. The angular sensitivity is roughly symmetrical about *θ* = 45° as output becomes dominated by FBG2 for *θ* > 45°.

In the Type II Rosette, the modulated energy output was dominated by the FBG1′s spectrum when *θ <* 45°. For a tensile stress signal, FBG1spectrum will move to the right and this effect alone should increase the modulated energy output. However, this movement will also increase its overlap with the FBG2 spectrum, resulting in a drop in modulated energy output. The combined effect results in a weaker increase in modulated energy output from the rosette than that from a single FBG1. For a compressive stress, FBG1 spectrum will move to the left and tends to move outside of the filter spectrum and will decrease the modulated energy output, leading to a decrease in energy. Again, this decrease will be counteracted as the movement increases the separation between the two FBG spectra, which will tend to increase the energy output. As a result, the decrease in output from the rosette will again be weaker than that from a single FBG1. Thus, for *θ* < 45°, the Type II Rosette output amplitude is significantly lower than that of the single FBG. On the other hand, if the output is dominated by FBG2, shifting the FBG2 spectrum will have reinforced combined effects and so the rosette output is stronger than that from a single FBG, as is evident in Figure 8 for *θ* > 45°.

By properly increasing the separation of the FBG Bragg spectra and positioning them on either side of one edge of the filter spectrum (Figure 3c), the signal weakening effect in the Type II Rosette for *θ* < 45°can be transformed into a strengthening effect. On the other hand, the strengthening effect for *θ* > 45° is retained. Thus, the Type III Rosette gives a strong and fairly even output for different *θ*’s. This rosette configuration is adopted for latter impact localization testing.

### 3.3. Impact Source Localization

It is clear from Section 2.2.3 that precise determination of the arrival times of the stress waves at each individual Fiber Bragg grating (FBG) is important to the performance of time difference of arrival-based impact localization schemes. It has been pointed out that error in a determined arrival time can be caused by (i) intrinsic noise of the measurement system and (ii) directional sensitivity of the FBG [16]. Due to dispersion and attenuation of Lamb wave, the amplitude of the leading waves is usually small and can be masked by the former. It is possible to be overcome by using a stronger light source and steeper edge filter to increase signal-to-noise ratio (SNR). For the latter, it may be improved by replacing each single FBG sensor with an FBG rosette proposed in this paper.

**Figure 9:**
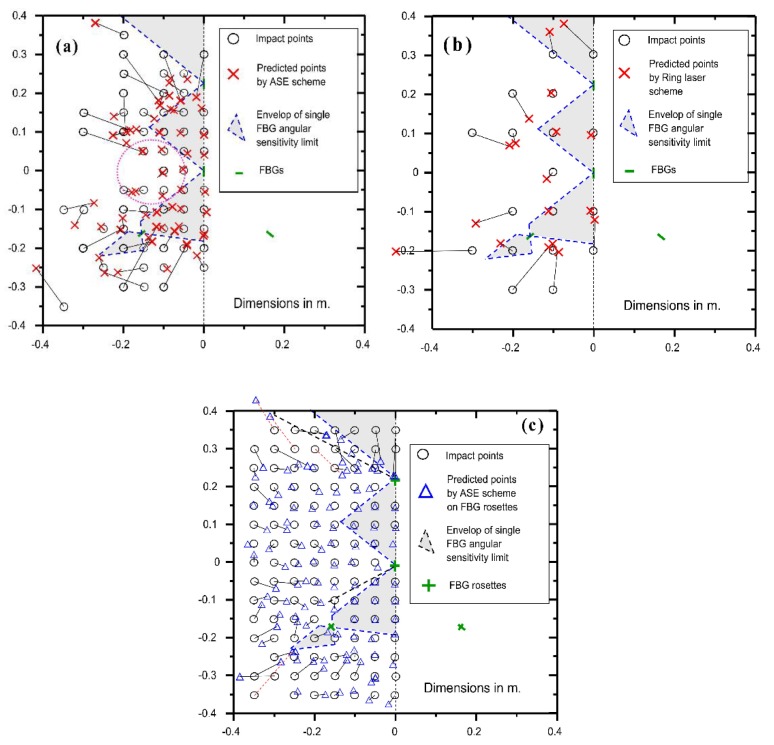
Comparison of the actual and deducted impact locations on the aluminum plate using (**a**) the amplified spontaneous emission (ASE) scheme/Single sensors; (**b**) the ring laser scheme/Single FBG sensors; (**c**) the ASE scheme/FBG rosette sensors.

To differentiate the relative effect of signal strength and directional sensitivity, impact source location test was first done with the amplified spontaneous emission scheme using single FBG sensors, as laid out in Figure 2. The test was carried out again with the above setting but with the interrogation scheme changed into the ring laser scheme. A third test employed the amplified spontaneous emission (ASE) scheme while the single FBG sensors were replaced with the Type III Rosette configuration. The impact localization results for these three tests are shown in Figure 9a to c, respectively. To help perceive the localization error, corresponding pairs of deduced and actual positions are connected by a straight line, except for those in obvious correlation. The positions of the sensors are also indicated in the figures to give an idea of the directionality limitation. Previous work showed that when an impact occurs at a location 50° or further off the FBG longitudinal axis, the arrival time identification would be seriously affected by the system noise. The dotted lines in Figure 9 extend along each FBG’s ± 50° off-axis direction. The region within the ± 50° boundaries of all the four sensors is shaded in grey, which includes two triangles and two quadrilaterals.

Figure 9a shows the impact localization results using the single FBG sensor array with the ASE interrogation scheme. The impacts within the middle grey triangle and quadrilateral regions could be accurately estimated. For those in the upper grey triangle and lower grey quadrilateral, the estimations have considerable error. This may be caused by the combination of two effects: (1) the regions are too far away from the sensors, so the arrival time detection for the attenuated stress waves was less accurate; (2) the incidence angle of the stress waves toward some of the sensors are large, so FBG’s directional sensitivity had a negative influence. Outside the grey shaded regions, estimations are not good in general, and for some points close to the plate edges, no convergent solution can be obtained. Except for some typical example data, points with a large discrepancy between the actual and predicted locations are not shown for clarity of presentation. Some exceptions are points inside the dotted circular region where reasonable estimations are obtained. Such points are either not too distant from the FBGs or close to the FBGs’ ± 50° off-axis boundaries. The former ensures less signal attenuation and hence a better signal-to-noise ratio. The latter implies that the directional sensitivity of the FBG may somewhat extend beyond 50° a bit. All in all, the above phenomena suggest that both the signal strength and the FBG’s directional sensitivity are important to give a good impact location prediction.

Figure 9b shows some typical prediction results when the single FBG array was interrogated by the ring laser scheme. If we compare the corresponding points in Figure 9a,b, it can be seen that the prediction error based on the ring laser scheme is in general smaller than that on the ASE scheme. This is reasonable, as the higher interrogation sensitivity and SNR of the former are expected to alleviate the problems of signal attenuation and background noise. However, the overall improvement is only marginal. As pointed out before in Figure 6, the lack of sensitivity when the signal source is in the 90° direction with respect to the FBG axis persisted even with the ring laser scheme. The above comparison suggests that the bottleneck of location accuracy is the directional sensitivity of an FBG. The underlying cause of FBG’s insensitivity at certain directions is rooted from the way an impact stress wave interacts with an FBG and this cannot be rectified with a more intense light source and/or steeper filter slope. Nevertheless, the ring laser scheme is advantageous as it allows either a single source to feed more sensors or the same sensor array to monitor impact over a larger area.

On the other hand, good correlation over the whole plate is obtained with the FBG rosette array, even when the lower intensity ASE scheme was used (Figure 9c). The worst-case errors between the deduced and actual impact positions are barely above 10 cm and these are indicated with broken straight lines. With the previous two schemes, the same positions mostly have much larger errors or even without convergent solutions. The above results show that reducing FBG’s directionality is much more effective to boost impact localization accuracy, even under the low intensity ASE light source.

**Figure 10:**
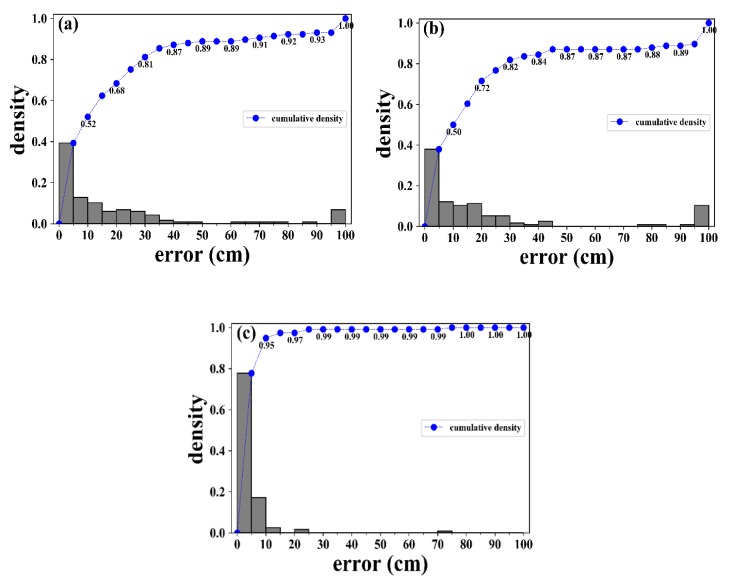
Distribution of the impact localization errors on the aluminum plate using (**a**) the amplified spontaneous emission (ASE) scheme/Single Fiber Bragg grating (FBG) sensors; (**b**) the ring laser scheme/Single FBG sensors; (**c**) the ASE scheme/FBG rosette sensors.

Figure 10 shows the histograms and cumulative density of the impact localization errors distributions. Each histogram has 20 bins, each with an equal bin size of 5 cm. The density of a bin represents the normalized frequency of the impact points having errors within the bin. The impact points with errors larger than 100 cm or those without convergent solutions were counted in the last bin (95 cm, 100 cm).

Figure 10a shows that around 40% and 50% of the errors were respectively smaller than 5 cm and 10 cm by using the single FBG sensor array with the ASE interrogation scheme. As depicted in Figure 9a, impact points with larger errors were either far away from the sensors or having large incidence angles of the stress waves toward some sensors.

Figure 10b shows that the ring laser scheme has similar proportion of errors between 0 cm and 10 cm. The slightly higher population with errors smaller than 20 cm and 30 cm (0.72 and 0.82) might demonstrate its marginal improvement over the ASE scheme (0.68 and 0.81) by reducing errors at some typical impact points. However, the ring laser scheme has heavier density in the last bin. It seems a combination of significantly enhanced sensitivity for smaller incidence angles and unchanged insensitivity in near 90° directions worsened the results for some impact positions.

Figure 10c shows clear superiority by using FBG rosettes where most of the errors were smaller than 10 cm. Larger errors only occurred in a few impact points far away from the sensors, and the overall result is much better than the single FBG cases.

## 4. Conclusions

A comprehensive review on impact localization schemes by using Fiber Bragg grating (FBG) sensors showed that FBG’s directional sensitivity could undermine the impact localization accuracy in general. The negative effect induced by FBG’s directionality was practically exemplified by localizing impacts on an aluminum plate by a time difference of arrival based method using a 4-FBG array and an amplified spontaneous emission (ASE) interrogation scheme. Enhancing the signal-to-noise ratio of the measurement systems four-fold by using a ring laser interrogation scheme only marginally improved the location accuracy, confirming the bottleneck of impact source localization accuracy is on the limitation of the insensitivity of an FBG over certain directions. To address this issue, rosettes consisting of two orthogonally cascaded FBGs have been proposed to replace the single FBG. The directional sensitivity of three FBG rosette configurations with different relative Bragg wavelengths has been investigated and it was found that the Type III rosette configuration shows nearly omni-directional sensitivity. Using Rosette III to replace each single FBG could significantly expand the area with quality impact localization accuracy to all over the aluminum plate, even though the lower intensity ASE scheme was used.

The proposed FBG rosettes can potentially be applied to improve the accuracy for most impact localization schemes. They may be realized inside fiber reinforced polymer composites by embedding two orthogonal FBGs respectively to adjacent 0° and 90° plies.
